# Leucine-rich α-2-glycoprotein promotes TGFβ1-mediated growth suppression in the Lewis lung carcinoma cell lines

**DOI:** 10.18632/oncotarget.3557

**Published:** 2015-03-12

**Authors:** Norihiko Takemoto, Satoshi Serada, Minoru Fujimoto, Hiromi Honda, Tomoharu Ohkawara, Tsuyoshi Takahashi, Shintaro Nomura, Hidenori Inohara, Tetsuji Naka

**Affiliations:** ^1^ Department of Otorhinolaryngology-Head and Neck surgery, Osaka University Graduate School of Medicine, Ibaraki, Osaka, Japan; ^2^ Laboratory of Immune Signal, National Institute of Biomedical Innovation, Ibaraki, Osaka, Japan; ^3^ Department of Animal Bioscience, Nagahama Institute of Bio-Science and Technology, Ibaraki, Osaka, Japan; ^4^ Department of Surgery, Osaka University Graduate School of Medicine, Osaka, Japan

**Keywords:** Leucine-rich α-2-glycoprotein, Lewis lung carcinoma, TGFβ, apoptosis, smad signal transduction

## Abstract

Leucine-rich α2-glycoprotein (LRG) is an approximately 50-kDa glycoprotein that has been found to be elevated in the sera of patients with several types of cancer. LRG directly binds to transforming growth factor beta 1 (TGFβ1) and modulates TGFβ1 signaling in endothelial cells; however, the precise function of LRG in cancer remains unclear. This study aimed to investigate the role of LRG in cancer. Lewis lung carcinoma (LLC) cells hardly expressed LRG. The growth of LLC tumors allografted in the LRG knockout (KO) mice was significantly increased compared with wild-type (WT) mice. Conversely, overexpression of LRG significantly inhibited the growth of LLC tumors in WT mice. In the presence of LRG, TGFβ1 significantly inhibited the proliferation of LLC cells and human hepatocellular carcinoma Hep3B cells *in vitro* by inducing apoptosis *via* the potent activation of smad2 and its downstream signaling pathway. Furthermore, administration of a TGFβR1 inhibitor (SB431542) significantly enhanced the growth of LLC tumors in WT mice compared with LRG KO mice *via* inhibition of apoptosis. We propose that LRG potentiates the effect of TGFβ1 in cancer cells whose growth is suppressed in the presence of TGFβ1.

## INTRODUCTION

Leucine-rich α2-glycoprotein (LRG) was first identified from human serum in 1977 [1]. LRG is an approximately 50-kDa glycoprotein that contains repetitive sequences with a leucine-rich motif [2]. This family of proteins, characterised by the presence of leuicine-rich repeats in their amino acid sequences, has been shown to be involved in protein–protein interactions, signal transduction, and cell development [3]. LRG expression is reportedly upregulated during early neutrophil granulocyte differentiation [4] and is increased in hepatocytes in response to the mediators of acute-phase response [5]. In a recent study, we demonstrated that the expression of LRG was elevated in the sera of patients with rheumatoid arthritis, Crohn's disease, and ulcerative colitis in the high disease activity state [6, 7]. Moreover, it has been shown that LRG is highly expressed in the sera of patients with bacterial infections [8]. In addition, serum LRG levels have been reported to be elevated in patients with several types of cancer, including lung [9, 10], ovarian [11], and biliary tract cancers [12]. Our group has shown that elevated expression of LRG can be detected not only in sera but also in the tumor tissues of pancreatic cancer [13]. Although LRG has been analyzed as a biomarker in these disorders, the physiological role of LRG in cancer has not been fully elucidated.

LRG directly binds to transforming growth factor beta (TGFβ) [14]. Recently, Wang *et al.* reported that LRG modulates TGFβ1 signaling in endothelial cells, resulting in the promotion of pathogenic angiogenesis [15]. TGFβ1 is a highly pleiotropic cytokine known to inhibit the proliferation of epithelial and lymphoid cells [16], while also having a suppressive role in carcinogenesis. Cellular responses to TGFβ1 vary depending on the cell type [17]. In previous studies, a proportion of prostate, bladder, gastric, hepatocellular, and ovarian cancers showed sensitivity to TGFβ1 and their growth was inhibited by stimulation with TGFβ1 [18- 22]. However, it remains unclear whether LRG modulates the sensitivity of cells to TGFβ1 signaling in cancer.

In the present study, we demonstrate that the growth of the murine Lewis lung carcinoma (LLC) and human hepatocellular carcinoma Hep3B cell lines was suppressed by stimulation with TGFβ1. In addition, we show that TGFβ1-induced apoptosis was augmented in the presence of LRG in both cell lines *in vitro* as well as in LLC cells *in vivo*. The growth inhibition of LLC cells was enhanced by TGFβ1-smad2 signaling in the presence of LRG.

## RESULTS

### LRG showed tumor growth inhibitory effects *in vivo*

**Figure 1:**
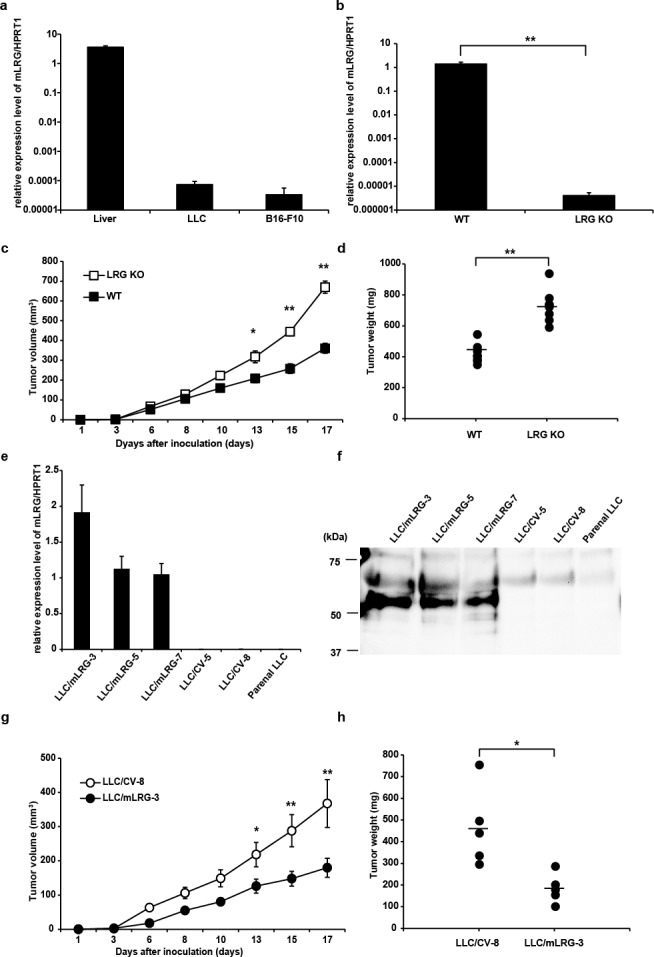
Tumor growth of LLC cells was inhibited in the presence of LRG *in vivo* a. Quantitative real-time PCR analysis of mLRG mRNA expression in mouse liver, LLC cells, and B16-F10 cells. Quantitative real-time PCR threshold values for target genes were normalized against the level of HPRT1. b. Quantitative real-time PCR analysis of mLRG mRNA expression in the LLC tissue of WT and LRG KO mice. Quantitative real-time PCR threshold values for target genes were normalized against the level of HPRT1. Each value is the average ± standard deviation. c. Tumor growth curves of LLC cells on subcutaneous injection into LRG KO mice or WT C57BL/6J mice (n = 8 for each group). d. Tumor weight on day 17 after implantation of LLC cells (n = 8). Data are presented as mean ± standard error of the mean. e,f. Western blot analysis of mLRG expression in the supernatant and quantitative real-time PCR analysis of mLRG mRNA levels in mLRG-overexpressing LLC clones (LLC/mLRG-3,5,7), control vector LLC clones (LLC/CV-5,8) and parental LLC cells. Quantitative real-time PCR threshold values for target genes were normalized against the level of HPRT1. Each value is the average ± standard deviation. g. Growth curves of control vector LLC (LLC/CV-8) and mLRG-overexpressing LLC (LLC/mLRG-3) cells implanted in WT C57BL/6J mice (n = 5 for each group). h. Tumor weight on day 17 after implantation of LLC/mLRG-3 and LLC/CV-8 cells (n = 5). Data are presented as mean ± standard error of the mean. * P < 0.05, **P < 0.01.

To investigate the expression levels of LRG in mouse cancer cell lines, LLC (mouse lung cancer) cells and B16-F10 (mouse melanoma) cells were analyzed using quantitative real-time polymerase chain reaction (PCR). We found that LLC and B16-F10 cells hardly expressed LRG compared with mouse liver tissue (Fig. 1a). To assess the effects of LRG on tumor growth, we used LLC cells to develop an allograft model. LLC cells were subcutaneously implanted into wild-type (WT) or LRG knockout (KO) mice, and tumor growth was evaluated. Quantitative real-time PCR revealed that the expression level of LRG in the implanted LLC tissue of WT mice was elevated, whereas LLC tissue in LRG KO mice expressed little LRG in LLC cells (Fig. 1b). Compared with WT mice, LRG KO mice showed a significant increase in the growth of LLC cells (Fig. 1c,d). To evaluate the effect of LRG produced by tumor cells *in vivo*, we established three stably mLRG-overexpressing clones (LLC/mLRG-3, -5, and -7) and two empty vector-transfected clones (LLC/CV-5 and -8) from parental LLC cells. Quantitative real-time PCR and western blot analyses confirmed the overexpression of mLRG. Expression of mLRG was hardly detected in parental LLC cells at both the mRNA and protein levels (Fig. 1e,f). To investigate the effect of LRG *in vivo*, an mLRG-overexpressing clone and an empty vector-transfected clone (LLC/mLRG-3 and LLC/CV-8, respectively) were implanted into WT mice, and tumor growth was evaluated in each group. As shown in Fig. 1g,h, overexpression of mLRG significantly inhibited the growth of LLC tumors.

### Overexpression of mLRG enhanced TGFβ1-induced growth inhibition in LLC cells

LRG is reportedly capable of binding to TGFβ1 [14]. Therefore, we quantitatively assessed the binding affinity of LRG and TGFβ1 by surface plasmon resonance (SPR) analysis using BIAcore 3000. As expected, binding between LRG and TGFβ1 was detected, and the equilibrium dissociation constant K_D_ was 2.32 μM (Supporting information Fig. S1), suggesting that LRG directly binds to TGFβ1.

**Figure 2:**
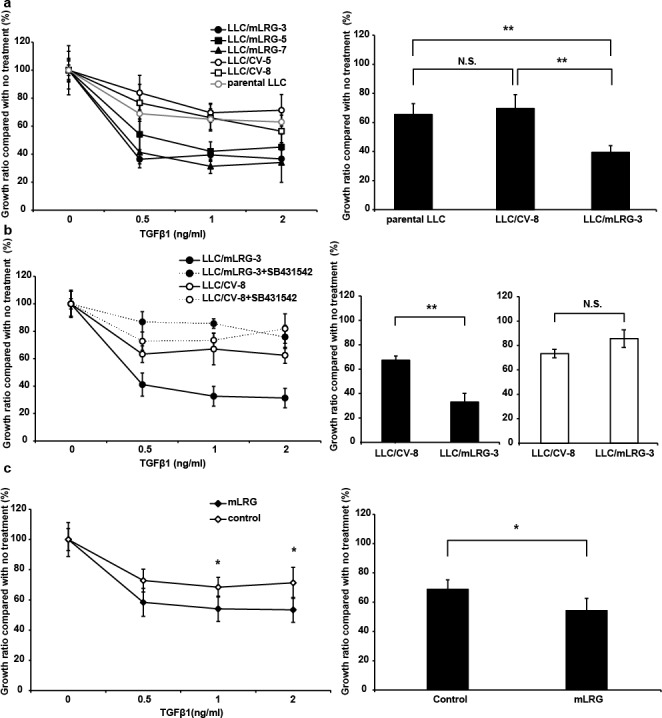
TGFβ1 inhibited the proliferation of mLRG-overexpressing LLC cells more effectively than that of control vector LLC cells a. The left panel shows the growth curves of mLRG-overexpressing LLC (LLC/mLRG-3,5,7), control vector LLC (LLC/CV-5,8), and parental LLC cells treated with TGFβ1. Cells were cultivated in the presence of TGFβ1 (0–2 ng/mL). After culture for 72 h, viable cell numbers were counted using the WST-8 assay. The right panel shows the viability of LLC/mLRG-3, LLC/CV-8, and parental LLC cells after treatment with 1.0 ng/mL of TGFβ1 for 72 h. b. The left panel shows the growth curves of mLRG-overexpressing LLC (LLC/mLRG-3) and control vector LLC (LLC/CV-8) cells treated with TGFβ1 and TGFβR1 inhibitor. Cells were cultured in the presence of TGFβ1 (0–2 ng/mL) with DMSO or a TGFβR1 inhibitor, SB431542 (10 μM). After culture for 72 h, viable cell numbers were counted using the WST-8 assay. The right panel shows the viability of LLC/mLRG-3 and LLC/CV-8 cells after treatment with 1.0 ng/mL of TGFβ1 with (black bar) or without (white bar) SB431542 (10 μM) for 72 h. c. The left panel shows the growth curves of parental LLC cells treated with TGFβ1 (0–2 ng/mL) combined with recombinant mLRG (10 μg/mL) from LLC/mLRG-3 cells or purified products from LLC/CV-8 cells. After culture for 72 h, viable cell numbers were counted using the WST-8 assay. Each value is the average ± standard deviation. The right panel shows the viability of parental LLC cells after treatment with 1.0 ng/mL of TGFβ1 for 72 h. Each value is the average ± standard deviation. * P < 0.05, **P < 0.01.

To investigate whether LRG modulates the effect of TGFβ1 on the proliferation of LLC cells, we compared the proliferation of three mLRG-overexpressing clones (LLC/mLRG-3, -5, and -7), two empty vector-transfected clones (LLC/CV-5 and -8), and parental LLC cells after stimulation with TGFβ1. The results of the 2-(2-methoxy-4-nitrophenyl)-3-(4-nitrophenyl)-5-(2,4-disulfophenyl)-2H tetrazolium, monosodium salt (WST-8) assay revealed that stimulation with TGFβ1 had a greater inhibitory effect on the proliferation of mLRG-overexpressing LLC clones than on empty vector-transfected clones and parental LLC cells (Fig. 2a). To confirm that these results were caused by enhancement of the effect of TGFβ1, LLC/mLRG-3 and LLC/CV-8 cells were treated with both TGFβ1 and SB431542, a TGFβR type1 kinase inhibitor. In the cell proliferation assay, SB431542 abrogated the inhibitory effect of TGFβ1 on the proliferation of these cell types equally (Fig. 2b). Parental LLC cells were treated with mLRG purified from the supernatant of mLRG-overexpressing LLC cells combined with TGFβ1. For control, we used purified products from the supernatant of control vector LLC cells. As shown in Fig. 2c, TGFβ1 combined with recombinant mLRG had a stronger inhibitory effect on the proliferation of parental LLC cells than it had on control cells. Thus, our data indicate that overexpression of mLRG enhanced the direct inhibitory effect of TGFβ1 on the proliferation of LLC cells.

### TGFβ1-induced apoptosis was strongly enhanced in mLRG-overexpressing LLC cells compared to control vector LLC cells

**Figure 3:**
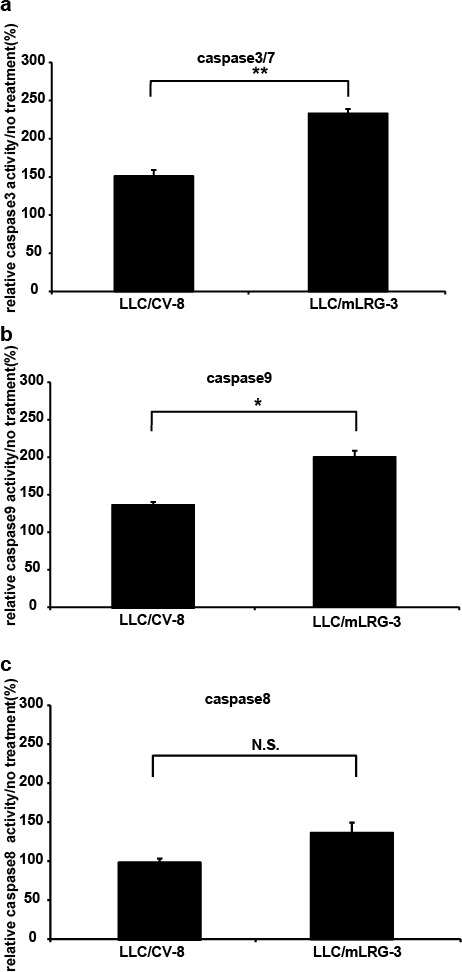
Stimulation with TGFβ1 induced apoptosis more strongly in mLRG-overexpressing LLC cells than in control vector LLC cells Caspase-3/7, caspase-8, and caspase-9 activities in LLC/mLRG-3 and LLC/CV-8 cells after treatment with TGFβ1. (a,b,c) Cells were cultivated in 96-well plates and treated with TGFβ1 (1.0 ng/mL) for 24 h. Caspase-3/7 activity was measured with a caspase-3/7 luminescent assay kit (Casapase-Glo^TM^). Similarly, caspase-8 and caspase-9 activities were measured using a caspase-8 and caspase-9 luminescent assay kit (Casapase-Glo^TM^). Each relative value (TGFβ1 treatment/no treatment) is the average ± standard deviation. * P < 0.05, **P < 0.01.

We next determined the effect of LRG combined with TGFβ1 on the apoptosis of LLC cells. To investigate TGFβ1-induced apoptosis of LLC/mLRG-3 and LLC/CV-8 cell lines, we measured caspase-3/7, caspase-8, and caspase-9 activities. As shown in Fig. 3a–c, the activities of caspase-3/7 and caspase-9, when stimulated with TGFβ1, were significantly increased in LLC/mLRG-3 cells compared with LLC/CV-8 cells. However, caspase-8 activity was not induced by TGFβ1 in either clone. These results indicate that LRG enhanced TGFβ1-induced apoptosis of LLC cells mainly through the intrinsic pathway.

### TGFβ1 induced the activation of smad2 and smad2 downstream signaling was significantly enhanced by overexpression of LRG

**Figure 4:**
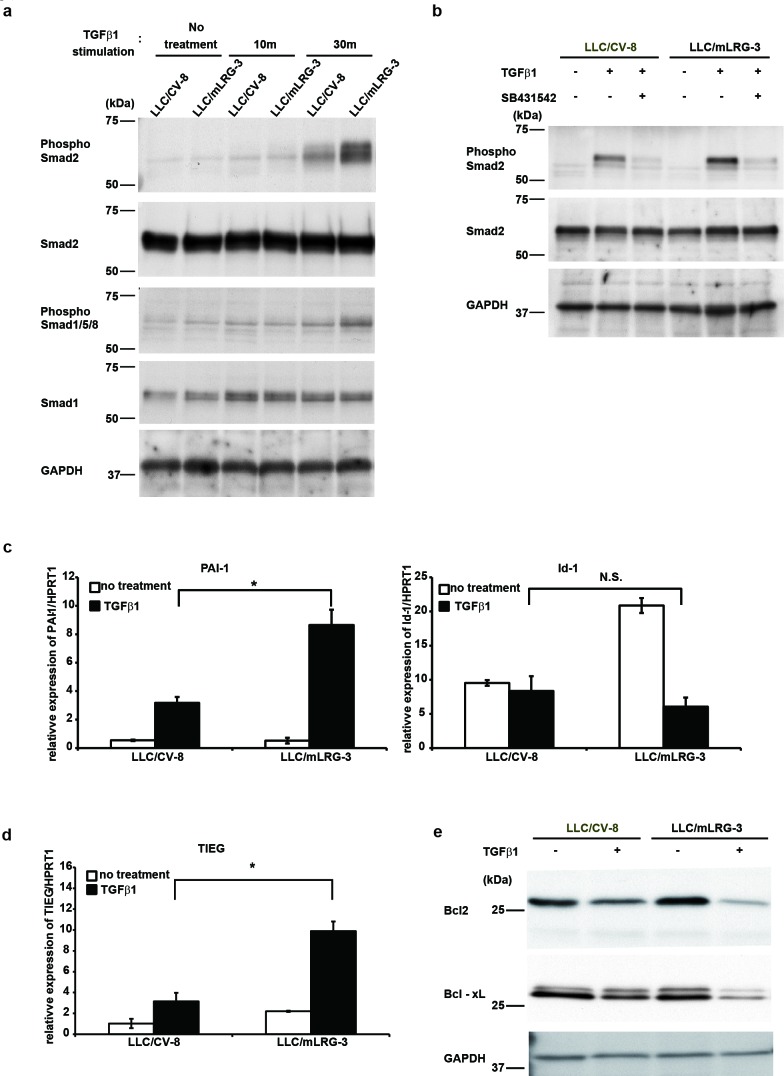
TGFβ1 enhanced the smad2 signaling pathway in mLRG-overexpressing LLC a. Western blot analysis shows the phosphorylation of smad2 and smad1/5/8 in LLC/mLRG-3 and LLC/CV-8 cells treated with TGFβ1. After 6 h of serum starvation, cells were treated with or without TGFβ1 (1.0 ng/mL) for 10 or 30 min. b. Western blot analysis shows the phosphorylation of smad2 in LLC/mLRG-3 and LLC/CV-8 cells treated with or without SB431542 (10 μM) and TGFβ1. Cells were treated with SB431542 (10 μM) or DMSO (vehicle) for 3 h and with TGFβ1 (1.0 ng/mL) for 30 min. c. Quantitative real-time PCR analysis shows PAI-1 gene and Id-1 gene expression with or without stimulation with TGFβ1 (1.0 ng/mL) for 3 h in LLC/mLRG-3 and LLC/CV-8 cells after 6 h of serum starvation. Quantitative real-time PCR threshold values for the target genes were normalized against the level of HPRT1. d. Quantitative real-time PCR analysis shows TIEG gene expression with or without stimulation with TGFβ1 (1.0 ng/mL) for 3 h in LLC/mLRG-3 and LLC/CV-8 cells after 6 h of serum starvation. Quantitative real-time PCR threshold values for the target genes were normalized against the level of HPRT1. e. Western blot analysis shows Bcl-2 and Bcl-xL protein in LLC/mLRG-3 and LLC/CV-8 cells treated with TGFβ1. Cells were cultured in 6-well plates with or without stimulation with TGFβ1 (1.0 ng/mL). After culture for 24 h, cell lysates were collected. Each value is the average ± standard deviation. *P < 0.01.

TGFβ1 regulates apoptosis *via* two distinct means: the smad-dependent pathway and the smad-independent pathway. Upon TGFβ1 stimulation, the smad2/3/4 complex activates the transcription of pro-apoptotic genes whose products are directly involved in the death pathway [17]. We therefore investigated the activation status of signaling molecules involved in TGFβ1-induced apoptosis of LLC cells. Our screening analyses indicated that the AKT, JNK, and p38 signaling pathways were not activated in LLC cells treated with TGFβ1 (data not shown). As shown in Fig. 4a, western blot analysis showed that the phosphorylation levels of smad2 were more strongly increased in mLRG-overexpressing LLC cells than in control vector LLC cells treated with TGFβ1. SB431542 abrogated the phosphorylation of smad2 by TGFβ1 in these cells equally (Fig. 4b). Consistent with these results, quantitative real-time PCR analysis showed that the expression of the plasminogen activator inhibitor-1 (PAI-1) gene, which is the transcriptional target gene of smad2/3, was stronger in mLRG-overexpressing LLC cells than in control vector LLC cells after treatment with TGFβ1. Conversely, the expression of the Id1 gene, which is the transcriptional target gene of smad1/5/8, was not enhanced in either of these cells (Fig. 4c). Next, we determined which of the genes regulated by smads mediated the pro-apoptotic effects of TGFβ1. TGFβ1-inducible early gene (TIEG) has been reported as a transcription product of smads that induces apoptosis by TGFβ1 in various epithelial cell types [23]. TIEG-induced apoptosis is known to be mediated by the downregulation of the Bcl-2 protein [24]. As shown in Fig. 4d,e, quantitative real-time PCR analysis showed that the expression of TIEG was significantly enhanced in mLRG-overexpressing LLC cells treated with TGFβ1 compared with control vector LLC cells. Western blot analysis also demonstrated that the expression of the anti-apoptotic proteins Bcl-2 and Bcl-xL in mLRG-overexpressing LLC cells was decreased after treatment with TGFβ1 compared with control cells. These data indicated that smad2/3 signaling enhanced the pro-apoptotic effects of TGFβ1 in mLRG-overexpressing LLC cells.

### LLC tumor growth inhibition in the presence of LRG was abrogated more effectively by the TGFβR1 inhibitor *in vivo*

**Figure 5:**
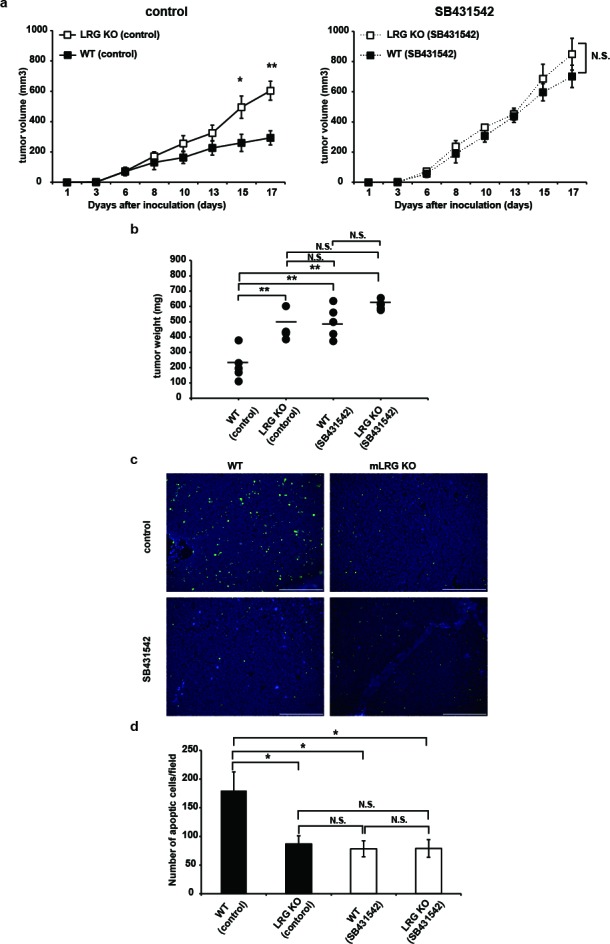
TGFβR1 inhibitor abrogated tumor growth inhibition in WT mice more effectively than that in LRG KO mice a. growth curves of LLC cells on subcutaneous injection into LRG KO mice or WT mice treated with SB431542 (10 mg/kg/week three times intraperitoneally) or vehicle control (n = 5 for each group). Solid lines represent control vehicle-treated groups and dashed lines represent SB431542-treated groups. b. Tumor weight of each group on day 17 after LLC cell inoculation (n = 5). c. TUNEL staining in LLC tumor sections from LRG KO or WT mice treated with SB431542 or vehicle control. Scale bar = 200 μm. d. Quantitative evaluation of TUNEL-positive cells in LLC tumors (five random fields of five independent tumor sections). Data are presented as mean ± standard error of the mean *P < 0.05, **P < 0.01.

To determine whether TGFβ is involved in LRG-mediated tumor growth inhibition *in vivo*, we evaluated the effect of SB431542 on the growth of LLC cells subcutaneously implanted into WT mice and LRG KO mice. SB431542 or a vehicle control was given by intraperitoneal (i.p.) injection three times a week after cell implantation. In the vehicle control-treated group, tumor growth was significantly enhanced in LRG KO mice compared with WT mice. On the other hand, in the SB431542-treated group, tumor growth curves were not significantly different between LRG KO mice and WT mice (Fig. 5a). On day 17, in the vehicle control-treated group, tumor weight of LRG KO mice was significantly higher than that of WT mice; however, no significant differences in tumor weight were found between LRG KO mice and WT mice in the SB431542-treated group (Fig. 5b). Furthermore, terminal deoxynucleotidyl transferase-mediated dUTP nick-end labeling (TUNEL) staining of LLC tumor tissue of the vehicle-treated group revealed the number of TUNEL-positive cells in LRG KO mice to be significantly lower than that in WT mice. In the SB431542-treated group, the number of TUNEL-positive cells was almost the same in LRG KO mice and WT mice (Fig. 5c,d). These results indicate that the inhibitory effect of the TGFβR1 inhibitor on the apoptosis of LLC tumors was enhanced in WT mice compared with LRG KO mice.

### LRG was involved in TGFβ1-induced apoptosis of Hep3B cell line

**Figure 6:**
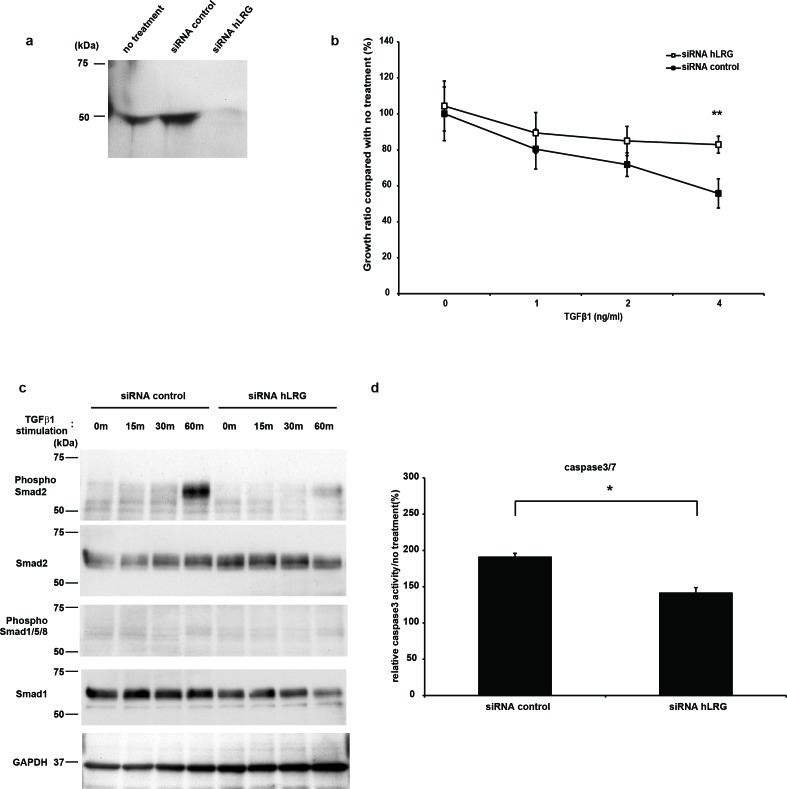
LRG was expressed in the supernatant of Hep3B, and TGFβ1-induced apoptosis was decreased in the absence of LRG a. Western blot analysis of hLRG expression in the supernatant of Hep3B. The supernatant was recovered from Hep3B cells transfected with hLRG siRNA or control siRNA for 24 h and expression of hLRG was assessed by western blot analysis. b. The growth curves of Hep3B with control siRNA and hLRG siRNA by stimulation with TGFβ1. After siRNA transfection, cells were cultivated in the presence of TGFβ1 (0–4 ng/mL). After culture for 72 h, viable cell numbers were counted using the WST-8 assay. c. Western blot analysis shows the phosphorylation of smad2 and smad1/5/8 in control siRNA or hLRG siRNA-transfected Hep3B cells treated with TGFβ1. After 4 h of transfection, cells were treated with TGFβ1 (4.0 ng/mL) for 0, 15, 30, or 60 min. d. Caspase-3/7 activity in control siRNA or hLRG siRNA-transfected Hep3B cells after treatment with TGFβ1. Cells were transfected with control siRNA or hLRG siRNA and subsequently treated with TGFβ1 (4.0 ng/mL) for 24 h in 96-well plates. Caspase-3/7 activity was measured using the caspase-3/7 luminescent assay kit (Casapase-GloTM). Each relative value (TGFβ1 treatment/no treatment) is the average ± standard deviation. **P < 0.01.

Hep3B cell line, which is derived from human hepatocellular carcinoma cells, has been reported to be sensitive to TGFβ1, and the cell growth of Hep3B was inhibited by treatment with TGFβ1 [20, 25- 27]. By western blot analysis, expression of LRG was observed in the culture supernatant of Hep3B cells; this expression was suppressed by transfection with small interfering RNA (siRNA) directed against LRG (Fig. 6a). We found that cell growth inhibition as well as caspase-3 activities of Hep3B cells *via* treatment with TGFβ1 were attenuated by transfection with LRG siRNA compared with control siRNA (Fig. 6b,c). In addition, the phosphorylation levels of smad2 induced by stimulation with TGFβ1 were also decreased when the expression of LRG was suppressed (Fig. 6d), suggesting that LRG enhanced TGFβ1-mediated smad2 signaling in Hep3B cells.

## DISCUSSION

In this study, we investigated the physiological functions of LRG in the TGFβ1-sensitive murine LLC cell line, which expresses low levels of LRG, by comparing the proliferation of LLC cells *in vitro* and *in vivo*. In a series of *in vitro* and allograft model experiments, the growth of LLC cells was significantly inhibited in the presence of LRG in a TGFβ1-dependent manner (Fig. 5a).

The TGFβ family plays a fundamental role in many cellular functions, including cell migration, survival, proliferation, and differentiation in a developmental context-dependent and cell type-specific manner. TGFβ1 reportedly often plays inhibitory roles [18- 20]; however, it has also been shown to promote cell proliferation [17, 28- 31]. It has been demonstrated that some proteins possessing leucine-rich repeats in their amino acid sequences can bind to TGFβ1 [32]. It has been suggested that LRG also binds to TGFβ1 in this manner [14]. Furthermore, we have confirmed that LRG directly binds to TGFβ1 by SPR analysis (Supporting information Fig. S1). Therefore, in this study, we tried to determine whether LRG can modulate the effects of TGFβ1 on TGFβ1-sensitive LLC cells. We showed that TGFβ1 inhibited the proliferation of LLC cells *in vitro*. Addition of TGFβ1 significantly inhibited the proliferation of LLC cells in the presence of mLRG, both in the case of mLRG-overexpressing cells and parental cells treated with recombinant mLRG purified from the supernatant. These results indicate that mLRG produced by mLRG-overexpressing LLC cells directly interacted with TGFβ1 in the extracellular environment and enhanced TGFβ1 signal transduction. In addition to LLC cells, we also demonstrated that TGFβ1-mediated cell growth suppression was enhanced by LRG in human hepatoma Hep3B cells, which have been reported to be sensitive to TGFβ1-mediated cell growth suppression, resulting in apoptosis (Fig. 6) [20, 25].

In our experiments using LLC and Hep3B cells, TGFβ1-stimulated activation of smad2 and its downstream signaling pathway was significantly increased in the presence of LRG. In previous reports on the Hep3B cell line, TGFβ1 induced apoptosis through the canonical smad2/3-4 signaling pathway [26, 33]. Connective tissue growth factor (CTGF) has been reported to be a secreted protein bound with TGFβ1. CTGF activates TGFβ1 signaling by direct binding in the extracellular space [34]. Therefore, we propose that similar to CTGF, LRG may be able to augment TGFβ1 signaling through binding to TGFβ1 in the extracellular space.

It has been reported that endoglin, an accessory receptor of TGFβ1, can switch from TGFβ1-smad2/3 signaling to TGFβ1-smad1/5/8 signaling in endothelial cells [35, 36]. LRG has been shown to enhance TGFβ1-smad1/5/8 signaling in the presence of endoglin in endothelial cells [15]. On the other hand, the activation of smad1/5 was reportedly attenuated, whereas the activation of smad2/3 was enhanced in the endothelial cells of endoglin heterozygote mice [37]. In human chondrocytes treated with endoglin siRNA, TGFβ1-induced smad2 phosphorylation was increased, but TGFβ1-induced smad1/5 phosphorylation was decreased [38]. We confirmed that the LLC and Hep3B cell lines expressed little or no endoglin compared with endothelial cells (Supporting information Fig. S2). Therefore, the lack of endoglin expression in LLC and Hep3B cells may implicate the promotion of TGFβ1-smad2 signaling instead of TGFβ1-smad1/5 signaling by LRG.

The TGFβR1 inhibitor significantly enhanced the growth and inhibited the apoptosis of LLC tumors in WT mice compared with LRG KO mice. These results demonstrated that LRG promoted TGFβ1-induced apoptosis *in vivo* as well as *in vitro.* In this study, we used LLC cells in which TGFβ1 had a growth-suppressive function; however, it has been reported that in many cell lines, TGFβ1-mediated growth suppression is disabled [39- 41]. Thus, in addition to LLC and Hep3B cells, we used B16-F10 cells (murine melanoma cell line) that do not express LRG (Fig. 1a). Stimulation of B16-F10 cells with TGFβ1 failed to inhibit proliferation *in vitro* (Supporting information Fig. S3). However, in a result similar to that obtained with LLC cells, promotion of tumor growth was observed in B16-F10 allograft model experiments using LRG KO mice (Supporting information Fig. S4). These results suggest that LRG *in vivo* has the growth-suppressive effect on tumors independently of its direct effect on TGFβ1 signaling. Instead, LRG may modulate other anti-tumor growth mechanisms *in vivo*, such as tumor immunity and tumor angiogenesis. Therefore, LRG may inhibit implanted LLC tumor growth by additional mechanisms other than enhancing TGFβ1 signaling. To reveal these indirect anti-tumor growth mechanisms *in vivo*, further analyses are required.

TGFβ1, which induces various cellular responses in a context-dependent manner, may function not only as a growth suppressor but also as a promotor of cancer depending on the disease stage [29, 30]. It has been reported that a glioblastoma cell line [42- 44] and an osteosarcoma cell line [40] proliferated by stimulation with TGFβ1 through smad2/3-dependent transcriptional regulation. Unlike the cell lines used in our investigation, growth of these cell lines, which show proliferation by TGFβ1 stimulation, could be promoted in the presence of LRG. Namely, LRG may enhance TGFβ1-smad signaling and accordingly enhance various cell type-specific and organ type-specific cellular responses to TGFβ1.

In conclusion, we found that in cells responsive to stimulation with TGFβ1 resulting in growth inhibition, induction of apoptosis by TGFβ1 was significantly enhanced in the presence of LRG, both *in vitro* and *in vivo*.

## METHODS

### Construction of the targeting vector for the LRG1 gene

A targeting vector was constructed to delete the coding sequences of exon 2 in the mouse *LRG1* gene. In brief, DNA fragments of the *LRG1* gene were obtained by PCR using BAC clones (RP23-180H19 or RP23-233P19) as templates. The amplified fragment for the long arm of the targeting construct was subcloned into a pBSII vector containing a diphtheria toxin A cassette (TK-DTA). The exon 2 fragment, together with a loxP site, was subcloned into a pBSIISK+ vector. The short arm fragment was subcloned into a pBSII vector containing a cassette with a loxP site adjacent to a flippase (FLP) recombinase target (FRT)-flanked neomycin-resistant gene (PGK-Neo). After enzymatic digestion of these vectors, the targeting vector was constructed by subcloning two inserts from the latter two vectors (the loxP-exon2 fragment and the Neo-loxP-short arm fragment) sequentially into the first vector containing a DTA cassette and the long arm.

### Generation of LRG-deficient mice

The targeting construct was linearized and electroporated into C57BL/6 embryonic stem (ES) cells. ES clones resistant to G418 were selected and screened for homologous recombination using PCR. After Southern blot analysis, a correctly targeted clone was injected into Balb/c blastocysts to generate chimeric mice, which were then mated with C57BL/6J mice to confirm germline transmission. Heterozygous mutant mice were selected using PCR and were crossed with C57BL/6J Cre-ER transgenic mice [45] obtained from the Jackson Laboratory (Stock number 004682). To delete the floxed *LRG1* gene, tamoxifen (2 mg/body, Sigma) was injected intraperitoneally into pregnant mice to induce Cre-ER-mediated gene recombination in embryos. The pups obtained were screened using PCR, and a pup with successful recombination was mated with C57BL/6J mice to obtain more LRG^+/−^ progenies. These LRG^+/−^ progenies were intercrossed to generate LRG-deficient mice. Deletion of the floxed LRG1 gene was identified by PCR genotyping using primers 5′-TACAGAAGAATTCCTGTTCACCTG-3′ and 5′-AGACGTGTCAAAGCCAGATAAACAC-3′

### Cell lines

Mouse LLC cells were obtained from the Japanese Collection of Research Bioresources (Osaka, Japan). Human hepatoma Hep3B cells were obtained from the Cell Resource Center for Biomedical Research (Tohoku University, Sendai, Japan). LLC and Hep3B cells were maintained in Dulbecco's modified Eagle's medium (DMEM) (Wako Pure Chemical Industries, Osaka, Japan) supplemented with 10% fetal bovine serum (FBS) (HyClone Laboratories, Logan, UT, USA) and 1% penicillin–streptomycin (Nacalai Tesque, Kyoto, Japan) at 37 °C under a humidified atmosphere of 5% CO_2_.

### Generation of mLRG stably transfected cell lines

To generate cell lines that stably expressed mLRG, LLC cells were transfected with the pcDNA3.1–mLRG-V5/His expression vector, as described previously [46]. Transfected cells were selected using 1000 μg/mL of geneticin (Invitrogen). Clones were maintained in 250 μg/mL of geneticin for stability of expression. Three stable mLRG-expressing cell lines were established and designated LLC/mLRG-3, LLC/mLRG-5, and LLC/mLRG-7. Two control cell lines of LLC cells stably transfected with an empty vector were also established. These cell lines were designated as LLC/CV-8 and LLC/CV-5.

### Recombinant mLRG preparation

Supernatants of both mLRG-expressing LLC cells and control vector LLC cells were harvested at 24 h from cultures (DMEM with 1% FBS and 250 μg/mL of geneticin) at a cell density of 1 × 10^6^ cells/mL. The harvested supernatants were purified using V5-tagged protein purification kit (Medical & Biological Laboratories, Nagoya, Japan) according to the manufacturer's instructions. Protein concentrations were determined using the DC protein assay kit (Bio-Rad Laboratories, Hercules, CA) using BSA as the concentration standard.

### Western blot analysis

Whole-cell protein extract was prepared from LLC or Hep3B cells in RIPA buffer [10 mmol/L Tris-HCl (pH 7.5), 150 mmol/L NaCl, 1% (v/v) NP-40, 0.1% (w/v) SDS, 0.5% (w/v) sodium deoxycholate, 1% protease inhibitor cocktail (Nacalai Tesque), and 1% phosphatase inhibitor cocktail (Nacalai Tesque)]. The extracted proteins were resolved on SDS–PAGE and transferred to an Immobilon-P transfer membrane (Millipore, Bedford, MA). The following antibodies were used: anti-phospho-Smad2 (Ser465/467) (1:1000), anti-Smad2 (D43B4) (1:1,000), anti-phospho-Smad1 (Ser463/465)/Smad5 (Ser463/465)/Smad8 (Ser426/428), (1:1,000), anti-Smad1 (D59D7) (1:1,000), anti-Bcl-xL (54H6) (1:1000), anti-Bcl-2 (50E3) (1:1,000) (all from Cell Signaling Technology, Danvers, MA); anti-GAPDH (1:1,000) (Santa Cruz Biotechnology, Santa Cruz, CA); anti-mLRG332 (1:500) (from IBL); and anti-human LRG (hLRG) polyclonal antibody (1:500) (from Proteintech Group, Chicago, IL). This was followed by treatment with 1:5,000 diluted donkey anti-rabbit horseradish peroxidase-conjugated secondary antibodies (GE Healthcare Bio-Sciences, Piscataway, NJ) and visualization using the Western Lightning ECL reagent (Perkin-Elmer, Boston, MA).

### Quantitative real-time and direct reverse transcriptase PCR of mRNA

Total RNA was isolated from LLC cells using the RNeasy Mini kit (Qiagen, Tokyo, Japan) according to the manufacturer's protocol. First, 100 ng of RNA was reverse transcribed using the QuantiTect reverse transcription kit (Qiagen). For quantitative real-time reverse transcriptase PCR, standard curves for mLRG, PAI-1, inhibitor of DNA binding-1 (Id-1), TIEG, and hypoxanthine phosphoribosyltransferase 1 (HPRT1) were generated from serial dilutions of positively expressing cDNA. Relative quantification of the PCR products was performed using ABI prism 7700 (Applied Biosystems, Darmstadt, Germany) and the comparative threshold cycle (CT) method. The level of the target gene expression was normalized to that of HPRT1 in each sample. The primers used for real-time PCR were as follows: mLRG, sense 5′-GGAGCAGCTATGGTCTCTTG-3′, antisense 5′-AGTATCAGGCATTCCTTGAG-3′; TIEG, sense 5′-AAGAACCCACGGAAATGTTG-3′, antisense 5′-GAGGAAGGCACAGCAAAGTC-3′; PAI-1, sense 5′-TCTTGCATCGCCTGCCAT-3′, antisense 5′-GGACCTTGAGATAGGACAGTGCTT-3′; Id-1, sense 5′-AGCCCTTCAGGAGGCAAGAG-3′, antisense 5′-GCGGTAGTGTCTTTCCCAGAGAT-3′; and HPRT1, sense 5′-TCAGTCAACGGGGGACATAA-3′, antisense 5′-GGGGCTGTACTGCTTAACCAG-3′. Each reaction was performed in triplicate. The variation within samples was less than 10%.

### siRNA transfection

siRNA treatment was performed with Dharmacon ON-TARGETplus SMART pool siRNA probes (Dharmacon) using ON-TARGETplus siCONTROL Nontargeting Pool (Dharmacon; D-001810-10) as a control. ON-TARGETplus SMART pool siRNA for hLRG (Dharmacon; L-015179-01-0010) was used. Cells were transfected with siRNA using Lipofectamine 2000 reagent (Invitrogen) according to the manufacturer's instructions.

### WST-8 assay

Parental LLC, mLRG-overexpressing LLC, and control vector LLC or Hep3B cell lines were plated in 96-well plates at a density of 2 × 10^3^ cells per well for LLC lines or 1.5 × 10^3^ cells per well for Hep3B lines in DMEM containing 10% FCS overnight. Thereafter, the medium was exchanged with DMEM containing 1% FCS in the presence of TGFβ1 (PeproTech, Rocky Hill, USA) and incubated for 72 h. In the case of Hep3B cells, hLRG siRNA or control siRNA was transfected and the medium was exchanged with DMEM containing 1% FCS in the presence of TGFβ1 and incubated for 72 h. In the experiment using TGFβ1 combined with recombinant mLRG purified from the supernatant of mLRG-expressing LLC cells, parental LLC cells were treated with purified mLRG and diluted to 10 μg/mL using DMEM containing 1% FCS. The purified supernatant of control vector LLC cells diluted at the same dilution rate served as the control. SB431542 (Wako, Osaka, Japan), a TGFβR1 inhibitor, was dissolved in dimethyl sulfoxide (DMSO) (Nacalai Tesque). The cells were treated with SB431542 (10 μM) or DMSO (vehicle) for 3 h, and TGFβ1 was added in each well to achieve the respective concentrations. After culture for 72 h, cell proliferation was evaluated using the WST-8 assay (Cell Counting Kit-SF; Nacalai Tesque). WST color development was measured and analyzed using a microplate reader (Model 680, Bio-Rad Laboratories) at a wavelength of 450 nm, with a reference wavelength of 630 nm. Experimental conditions were tested in sextuplicate (six wells in the 96-well microplate per experimental condition), and three independent experiments were performed.

### Apoptosis assay

Caspase-3/7 activities were measured using the Caspase-Glo^®^3/7 assay kit (Promega, Madison, WI, USA) according to the manufacturer's instructions. In brief, mLRG-overexpressing LLC and control vector LLC cell lines were plated in 96-well plates at a density of 2 × 10^3^ cells per well in DMEM containing 10% FCS overnight; then, the medium was exchanged with DMEM containing 1% FCS with TGFβ1 (1.0 ng/mL) and incubated for 24 h. In the experiment using the Hep3B cell line, 1.5 × 10^3^ cells per well were plated in DMEM containing 10% FCS overnight. After hLRG siRNA or control siRNA was transfected, the medium was exchanged with DMEM containing 1% FCS in the presence of TGFβ1 (4.0 ng/mL) and incubated for 24 h. The cells were incubated for 1 h at 37 °C with equal volumes of Caspase-Glo^®^3/7 reagent and the culture medium. Luminescence proportional to caspase-3/7 activities was determined using a luminometer (SoftMax○RRPro Molecular Devices). Similarly, caspase-8 and caspase-9 activities were measured using the Caspase-Glo^®^8 assay kit. These assays were performed three times in sextuplicate.

### *In vivo* tumor cell allograft model and treatment with the TGFβR1 inhibitor

All animal experiments were conducted in accordance with the Institutional Ethical Guidelines for Animal Experimentation of National Institute of Biomedical Innovation (Osaka, Japan). WT C57BL/6J mice were obtained from Charles River Japan (Yokohama, Japan). Parental LLC tumor cells (1 × 10^7^ per mouse in 0.1 mL PBS) were subcutaneously implanted into the flanks of LRG KO or WT C57BL/6J mice (8 weeks of age, female, n = 8 per group). For the use of stably overexpressing LLC cells, mLRG-overexpressing LLC cells (LLC/mLRG-3) and control vector-transfected LLC cells (LLC/CV-8) (1 × 10^7^ per mouse in 0.1 mL PBS) were subcutaneously implanted into the flanks of WT mice (8–9 weeks of age, female, n = 5 per group).

Experimental treatment with the TGFβR1 inhibitor was performed as follows. Parental LLC cells (5 × 10^6^ per mouse in 0.1 mL PBS) were subcutaneously implanted into the flanks of LRG KO or WT mice (8 weeks of age, female). When tumors were palpable (3 days after implantation of the LLC cells), LRG KO and WT mice were divided into two groups (n = 5 per group) to receive either the vehicle or SB431542 (10 mg/kg). SB431542 dissolved in DMSO was diluted 1:5 using corn oil (Sigma-Aldrich). The vehicle control was DMSO diluted in corn oil. Tumor volumes were determined three times weekly by measuring length (L), width (W), and depth (D). Tumor volume was calculated using the formula: tumor volume (mm^3^) = W × L × D. At 17 days after tumor implantation, tumors were removed and weighed. These three experiments were performed independently.

### TUNEL staining

Tumors were harvested and embedded in paraffin for immunohistochemical analysis. TUNEL assay [with 4′,6-diamidino-2-phenylindole (DAPI) nuclear counterstaining] for apoptosis was performed using the ApopTag^®^ fluorescein in situ apoptosis detection kit (Chemicon International, Temecula, CA) according to the manufacturer's instructions. Tumor sections were observed and images were acquired using Biozero BZ-9000 (Keyence, Tokyo, Japan). The number of positive cells in the sections was calculated using the BZ-II analyzer (Keyence). In all analyses, positive cells were quantified from five random fields.

### Statistical analysis

Statistical analyses were performed using one-way ANOVA followed by the Turkey–Kramer multiple comparison test to evaluate the significance of differences. When only two groups were compared, Student's t-test was used. In all analyses, P < 0.05 was considered to be statistically significant.

## SUPPLEMENTARY MATERIALS, FIGURES



## Abbreviations

DMEM: Dulbecco's modified Eagle's medium
FBS: fetal bovine serum
Id-1: inhibitor of DNA binding-1
LRG: leucine-rich α2-glycoprotein
LLC: Lewis lung carcinoma
PAI-1: plasminogen activator inhibitor-1
TGFβ: transforming growth factor beta
TIEG: TGFβ-inducible early gene
