# Supplementary material for: Leucine-rich α-2-glycoprotein promotes TGFβ1-mediated growth suppression in the Lewis lung carcinoma cell lines
Source: Oncotarget. 2015 Mar 12;6(13):11009–22. doi: 10.18632/oncotarget.3557 (PMC4484435; doi:10.18632/oncotarget.3557)
Supplement: Supplementary file 1 [file oncotarget-06-11009-s001.pdf]

Leucine-rich α-2-glycoprotein promotes TGFβ1-mediated growth suppression in the Lewis lung carcinoma cell lines

Supplementary Materials and Methods

Cell lines

Mouse melanoma cells (B16-F10) were obtained from the Japanese Collection of Research Bioresources (Osaka, Japan); mouse endothelial cells (MS-1) were obtained from ATCC (Manassas, VA). B16-F10 and MS-1 cells were maintained in DMEM (Wako Pure Chemical Industries, Osaka, Japan) supplemented with 10% FBS (HyClone Laboratories, Logan, UT, USA) and 1% penicillin–streptomycin (Nacalai Tesque, Kyoto, Japan). Human umbilical vein endothelial cells (HUVECs) were obtained from Lonza (Walkersville, U.S.). HUVECs were cultured in complete EGM-2 medium from Lonza. All cells were cultured at 37 °C under a humidified atmosphere of 5% CO_2.

Preparation of recombinant hLRG

Full-length hLRG C-terminal tagged with myc/His was transfected into CHO cells and hLRG was purified from the culture medium using a Ni-nitrilotriacetic acid column according to the manufacturer's instructions (Clontech). Purified hLRG was dialyzed using PBS.

SPR analysis

The binding affinity of each recombinant hLRG and TGFβ1 was assessed by SPR analysis using BIAcore 3000 (GE Healthcare UK Ltd., Chalfont, United Kingdom). hLRG was immobilized on CM5 BIAcore™ chips using an amine coupling kit according to the manufacturer's protocol (GE Healthcare). Next, various concentrations of recombinant TGFβ1 (PeproTech, Rocky Hill, USA) dissolved in HBS-P buffer (GE Healthcare) were injected into a biosensor chip at a flow rate of 20 µL/min for 2 min and SPR of the on-rate kinetics was monitored. SPR of the off-rate kinetics phase was monitored with the HBS-P buffer running over the sensor surface at 20 µL/min for 3 min. At the end of the off-rate kinetics phase, residual bound antibody was removed by flushing with 10 mM glycine–HCl (pH 3.0; BIAcore) at a flow rate of 20 µL/min for 0.5 min. Binding to the sensor chip is given as resonance units (RU). Data were analyzed by BIAevaluation 4.1 software (BIAcore).

***In vivo* B16-F10 tumor cell allograft model**

All animal experiments were conducted in accordance with the Institutional Ethical Guidelines for Animal Experimentation of National Institute of Biomedical Innovation

(Osaka, Japan). WT C57BL/6J mice were obtained from Charles River Japan (Yokohama, Japan). B16-F10 cells (1×10^6 per mouse in 0.1 mL PBS) were subcutaneously implanted into the flanks of LRG KO or WT C57BL/6J mice (8 weeks of age, female, n = 8 per group). Tumor volumes were determined three times weekly by measuring L, W, and D. Tumor volume was calculated using the formula: tumor volume (mm^3) = W × L × D. At 17 days after tumor implantation, tumors were removed and weighed. These three experiments were performed independently.

WST-8 assay

B16-F10 cell lines were plated in 96-well plates at a density of 1.5×10^3 cells per well in DMEM containing 10% FCS overnight; thereafter, the medium was exchanged with DMEM containing 1% FCS in the presence of TGFβ1 (PeproTech, Rocky Hill, USA) and incubated for 72 h. After culture for 72 h, cell proliferation was evaluated using the WST-8 assay (Cell Counting Kit-SF; Nacalai Tesque). WST color development was measured and analyzed with a microplate reader (Model 680, Bio-Rad Laboratories) at a wavelength of 450 nm, with a reference wavelength of 630 nm. Experimental conditions were tested in sextuplicate (six wells in the 96-well microplate per experimental condition), and three independent experiments were performed.

Western blotting

Whole-cell protein extract was prepared from LLC, MS-1, and Hep3B cells and HUVECs in RIPA buffer [10 mmol/L Tris-HCl (pH 7.5), 150 mmol/L NaCl, 1% (v/v) NP-40, 0.1% (w/v) SDS, 0.5% (w/v) sodium deoxycholate, 1% protease inhibitor cocktail (Nacalai Tesque), and 1% phosphatase inhibitor cocktail (Nacalai Tesque)]. The extracted proteins were resolved on SDS–PAGE and transferred to an Immobilon-P transfer membrane (Millipore, Bedford, MA). The following antibodies were used: anti-endoglin (MJ7/18) (1:1,000) for LLC and MS-1 cells and anti-endoglin (P4A4) (1:500) for Hep3B cells and HUVECs (Santa Cruz Biotechnology, Santa Cruz, CA). This was followed by a treatment with 1: 5,000 diluted HRP-conjugated goat anti-rat IgG secondary antibodies (Invitrogen, Carlsbad, CA, USA); they were visualized with using the Western Lightning ECL reagent (Perkin-Elmer, Boston, MA)

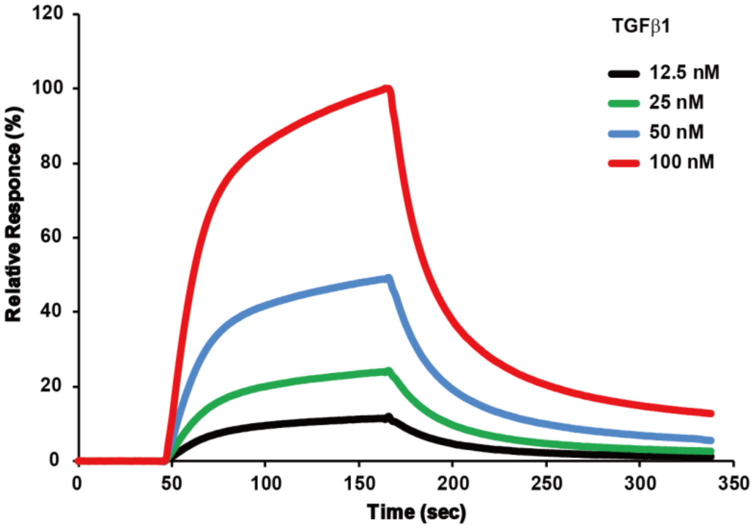

ka (1/Ms)	2.54×10^4
kd (1/s)	0.0589
K_D(μM)	2.32
Fit (X^2)	11.4

Supplemental Figure S1

The binding affinity of hLRG and TGFβ1 was analyzed using BIAcore. hLRG was immobilized on an CM5 sensor chip, and increasing amounts of TGFβ1 were applied. The interaction was normalized to the maximum observed response to get the relative response (%). The kinetic parameters of the binding reaction are shown under the sensorgrams.

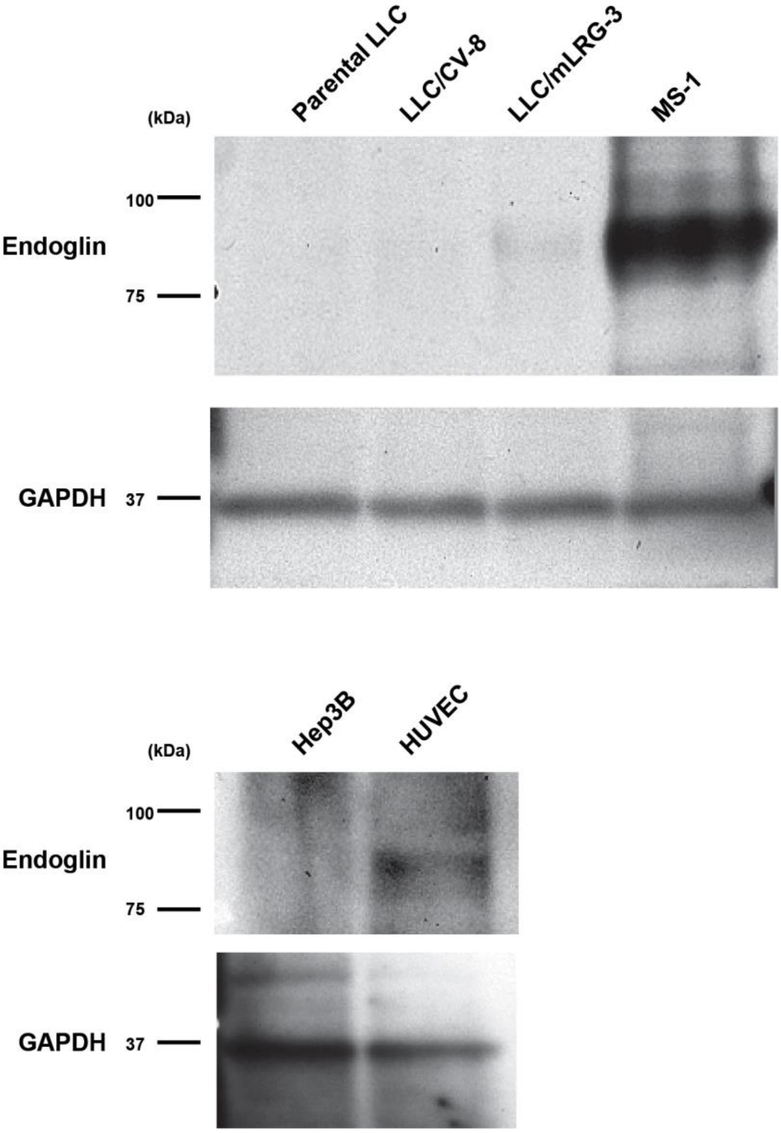

Supplemental Figure S2

Western blot analysis shows endoglin protein in parental LLC, LLC/mLRG-3, LLC/CV-8, MS-1, Hep3B cells and HUVECs. After the cells were cultured in 6-well plate for 24 h, cell lysates were collected.

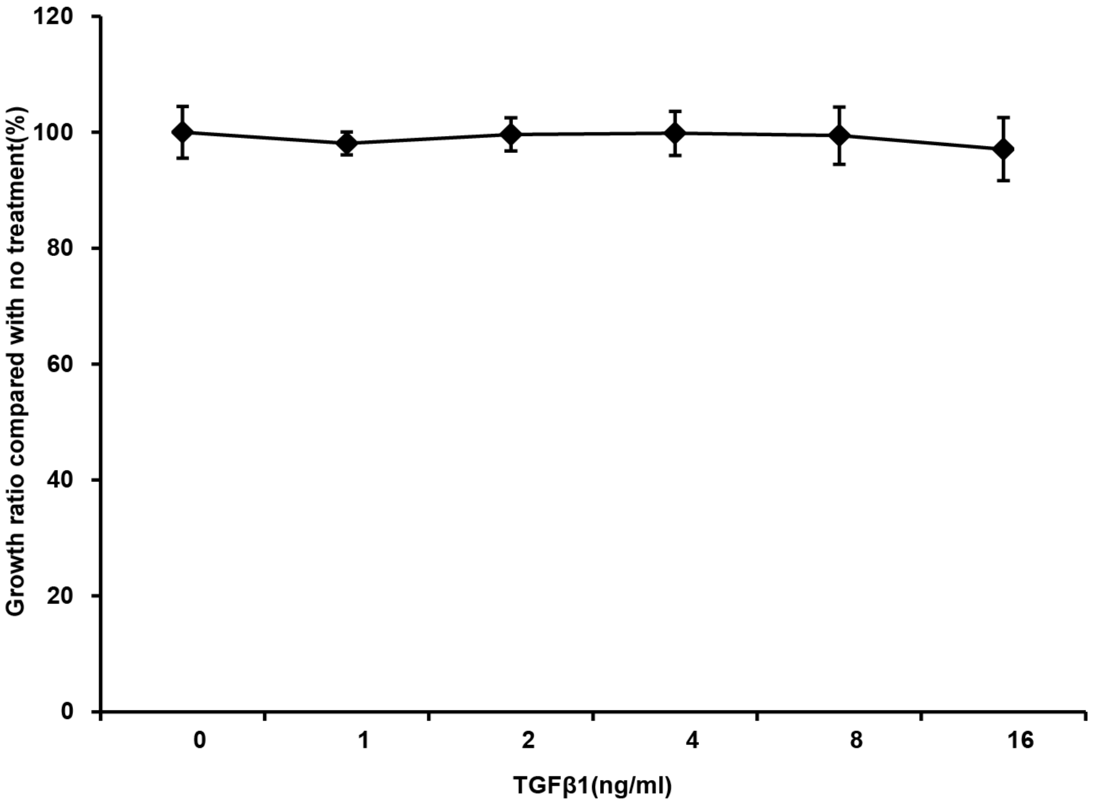

Supplemental Figure S3

The growth curves of B16-F10 cells treated with TGFβ1. The cells were cultivated in the presence of TGFβ1 (0–16 ng/mL). After culture for 72 h, viable cell numbers were counted using the WST-8 assay.

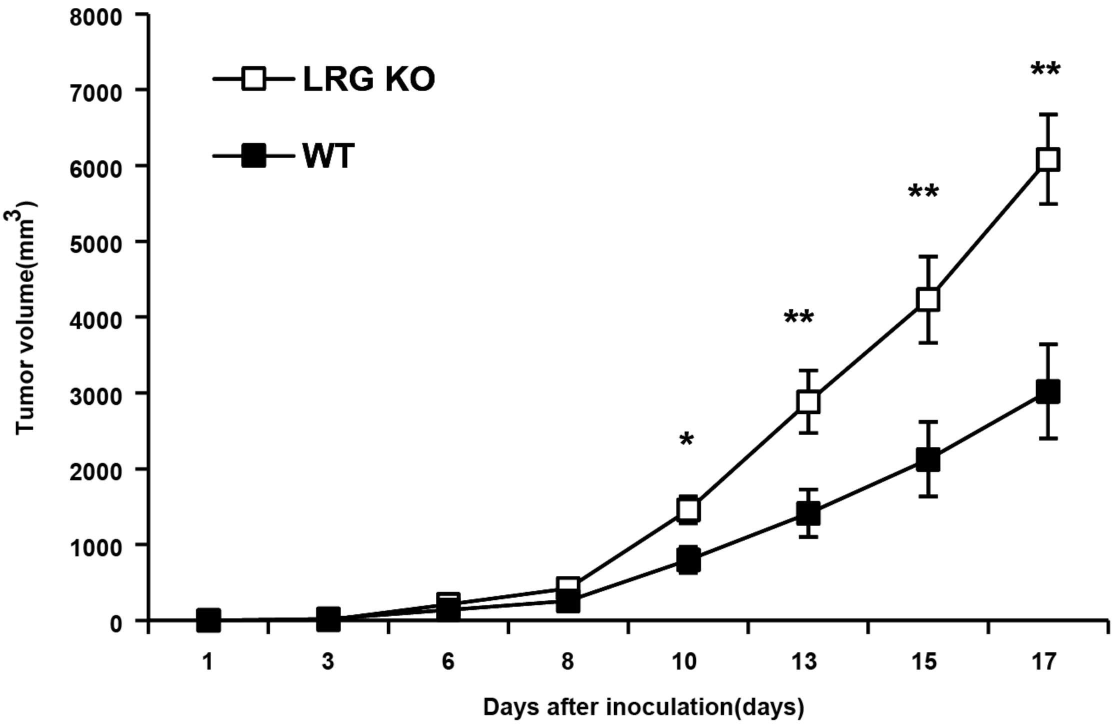

Supplemental Figure S4

Tumor growth curves of B16-F10 cells on subcutaneous injection into LRG KO mice or WT C57BL/6J mice (n = 8 for each group). Data are presented as mean ± standard error of the mean.
